# Identification of SWI/SNF Subcomplex GBAF Presence, Intra-Complex Interactions, and Transcriptional Dynamics during Early Porcine Development

**DOI:** 10.3390/ani14050773

**Published:** 2024-02-29

**Authors:** Sarah Innis, Aktan Alpsoy, Jennifer Crodian, Yu-Chun Tseng, Emily Dykhuizen, Birgit Cabot, Ryan Cabot

**Affiliations:** 1 Department of Animal Sciences, Purdue University, West Lafayette, IN 47907, USA; sinnis@purdue.edu (S.I.);; 2 Department of Medicinal Chemistry and Molecular Pharmacology, Purdue University, West Lafayette, IN 47907, USA

**Keywords:** epigenetics, developmental biology, embryo, gene regulation, trophoblast

## Abstract

**Simple Summary:**

Epigenetics is the study of heritable features known as epigenetic marks and other mechanisms that regulate how and when gene expression happens within a cell. Understanding the epigenetic status of cells during early embryonic and fetal development provides clues as to how development progresses under normal circumstances and a basis for comparison in cases where development does not progress as expected. Pigs are a useful organism for studying early development, as not only are they a staple livestock species, but they also have many physiological similarities to humans. This research identified the SWI/SNF chromatin remodeling complex GBAF in pig oocytes, embryos, and early development cell lines. Chromatin remodeling complexes can change how DNA is organized and packaged in a cell’s nucleus and influence gene expression as a result. GBAF, a key epigenetic mechanism, may regulate gene expression during early pig development by influencing how DNA is organized in a cell’s nucleus. Investigating these epigenetic controls is vital for understanding the complex network of factors responsible for shaping growth, development, reproductive efficiency, and overall animal health.

**Abstract:**

Understanding the complex interplay between genetics and environmental factors is vital for enhancing livestock production efficiency while safeguarding animal health. Despite extensive studies on production-specific genes in livestock, exploring how epigenetic mechanisms and heritable modifications govern animal growth and development remains an under-explored frontier with potential implications across all life stages. This study focuses on the GBAF chromatin remodeling complex and evaluates its presence during embryonic and fetal development in swine. Immunocytochemistry and co-immunoprecipitation techniques were employed to investigate the presence and interactions of GBAF subunits BRD9 and GLTSCR1 in porcine oocytes, preimplantation embryos, and cell lines, and transcriptional dynamics of GBAF subunits across these key developmental stages were analyzed using existing RNA-seq datasets. BRD9 and GLTSCR1 were identified across all represented stages, and an interaction between GLTSCR1 and BAF170 was shown in PTr2 and PFF cells. Our findings highlight the ubiquitous presence of GBAF in porcine early development and the potentially novel association between GLTSCR1 and BAF170 in swine. The transcriptional dynamics findings may suggest GBAF-specific contributions during key developmental events. This study contributes to the growing understanding of epigenetic regulators in both swine and mammalian development, emphasizing the implications of GBAF as a modulator of key developmental events.

## 1. Introduction

As the global population continues to rise amid numerous environmental challenges, addressing the increasing demand for food animal production necessitates enhancing efficiency and sustainability. Achieving this goal requires not only strategies to boost production output but also robust approaches to safeguard animal health and well-being. Understanding the intricate interplay between environment, genetics, and animal growth has gained prominence in both human and animal agriculture contexts. While studies in domestic livestock have primarily focused on identifying production-specific genes for efficient genomic selection, the exploration of heritable epigenetic modifications remains an under-explored avenue with potential implications for both phenotypic outcomes and animal health.

Epigenetic mechanisms, known to regulate various biological processes in humans and rodents, including growth, metabolism, development, and disease response [1,2,3], may hold comparable significance in livestock. Despite demonstrated implications across species, notable knowledge gaps persist concerning the epigenetic regulation of livestock development, performance, and health. In this context, studying chromatin remodeling complexes emerges as a crucial avenue for advancing both human health and animal agriculture.

Within the eukaryotic nucleus, chromatin, a condensed complex of DNA and proteins, plays a pivotal role in regulating gene expression. Chromatin remodeling complexes, particularly ATP-dependent complexes like SWI/SNF (for SWItch/Sucrose Non-Fermentable), modulate chromatin architecture, influencing transcriptional activation and repression. The aberrant function of these remodelers can interrupt embryonic development and promote disease progression [4,5]. Histone-modifying enzymes, such as acetylases and methylases, contribute to covalent histone modifications, impacting the accessibility of transcriptional machinery [6]. ATP-dependent chromatin remodeling complexes alter nucleosome positioning, regulating access to DNA [7,8].

Presently, there are four known eukaryotic chromatin remodeling complex families: ISWI, INO80, NuRD (also known as Mi-2 or CHD), and SWI/SNF. Chromatin remodeling complexes from these families are ATP-dependent, contain a similar central ATPase domain, and can be distinguished from one another based on their subunit composition [4]. Mammalian SWI/SNF chromatin remodeling complexes each contain a central ATPase (either BRG1 or BRM) and 10–15 accessory subunits [9]. The 2MDa mammalian BAF (BRM/BRG1 Associated Factor) subcomplexes are part of the SWI/SNF family of remodelers [9,10] and are distinguishable from one another due to the incorporation of unique constituent subunits. The recent identification of a smaller non-canonical BAF complex (ncBAF, or GBAF) in mouse and human cancer cell lines by Alpsoy and Dykhuizen showed that this BAF subcomplex is defined by the exclusion of several canonical BAF subunits and the inclusion of two unique subunits, bromodomain-containing 9 (BRD9) and mutually exclusive paralogs GLTSCR1 and GLTSCR1L (for glioma tumor suppressor candidate region gene 1 and gene 1 like) [11]. 

BAF complexes have known associations with pluripotency regulators such as OCT4, SOX2, and NANOG [12,13], and BRD9 is believed to contribute towards naïve pluripotency maintenance in mouse embryonic stem cells (ESCs) [14]. Furthermore, several links between BRD9 and tumorigenesis have been identified in cancers such as acute myeloid leukemia [15] squamous cell lung cancer (SqCLC) [16], malignant rhabdoid tumors (MRT) [17], and synovial sarcoma (SS) [18,19]. Both GLTSCR1 and GLTSCR1L are present in mouse ESCs [14], but little research has been done to explore the function of these subunits within the context of early mammalian development. GLTSCR1 is, however, linked to various cancer pathologies, including oligodendroglioma [20], prostate cancer [11,21], colorectal cancer [22], and SS [18]. Previous research has explored the developmental requirements of several BAF and PBAF subunits in mouse and swine embryonic contexts [23,24,25,26,27,28,29,30,31]. While research has worked to unravel the connections between GBAF subunits and cancer, their presence and function in early mammalian development, especially in large animal species like swine, remains largely unexplored.

As GBAF has been identified in other mammalian species, we hypothesize that this chromatin remodeling complex is also present in swine, including in the early stages of porcine development. To address this hypothesis, we aimed to identify BRD9 and GLTSCR1 in porcine oocytes, preimplantation embryos, and cell lines and examine the transcriptional dynamics of GBAF subunits across these key developmental time points. 

## 2. Materials and Methods

Unless otherwise stated, all chemicals were procured from Sigma Chemical Company, St. Louis, MO, USA. All cell culture reagents were obtained from ThermoFisher Scientific, Waltham, MA, USA.

### 2.1. Oocyte Collection and In Vitro Embryo Culture

Porcine ovaries were obtained from a local abattoir and brought back to the laboratory for aspiration subsequent oocyte and embryo culture as previously described [30]. Briefly, antral ovarian follicles were aspirated to collect follicular fluid. Germinal vesicle oocytes were obtained by denuding cumulus-oocyte complexes (COCs) collected from the follicular fluid using 0.1% hyaluronidase in 4-(2-hydroxyethyl)-1-piperazineethanesulfonic acid (HEPES)-buffered medium containing 0.01% polyvinyl alcohol. For in vitro embryo development, COCs were matured for 44 h at 39 °C, 5% CO_2_, and 100% humidity in Tissue culture medium 199 (TCM-199) supplemented as previously detailed [32]. Following maturation, cumulus cells were removed from matured oocytes. The denuded, matured oocytes were fertilized with fresh, extended boar semen according to previous methods [32]. Presumptive zygotes were cultured at 39 °C, 5% CO_2_, and 100% in porcine zygote medium 3 (PZM3) supplemented with 3 mg/mL BSA. Embryos at the 4-cell and blastocyst stages of development were collected at 48 h and 6 days post-gamete mixing, respectively. 

### 2.2. Cell Lines and Culture Conditions

Porcine fetal fibroblasts were grown as previously described [33]. Briefly, cells were cultured in Dulbecco’s Modified Eagle Medium (DMEM) supplemented with 15% FBS, 1% L-glutamine, 1% sodium pyruvate, 1% MEM-nonessential amino acids, and 1% penicillin-streptomycin. Non-primary porcine trophectoderm cells were grown as previously described [34] in phenol red-free DMEM-F12 supplemented with 5% FBS, 0.1 units/mL bovine insulin, 1% L-glutamine, and 1% penicillin-streptomycin. Both cell lines were cultured in 150 cm^2^ cell culture flasks at 39 °C, 5% CO_2_, and 100% humidity, and passaged at 90% confluency using trypsin/EDTA.

### 2.3. Nuclear Lysate Extraction

Cells were harvested by trypsinization and washed once in ice-cold phosphate-buffered saline (pH 7.2). The suspension was centrifuged at 400× *g* for 7 min at 25 °C. The pellet was resuspended in buffer A (20 mM HEPES, pH 7.9, 25 mM KCl, 10% glycerol, 0.1% Nonidet P-40), supplemented with a commercially available protease inhibitor cocktail (ThermoFisher), and incubated on ice for 7 min. Nuclei were isolated by centrifugation at 645× *g* for 10 min at 4 °C. Pelleted nuclei were resuspended in lysis buffer (50 mM HEPES, pH 7.5, 150 mM NaCl, 0.5% Nonidet P-40, supplemented with protease inhibitor), rotated at 4 °C for 30 min, then the extracts were cleared by centrifugation at 21,000× *g* at 4 °C for 30 min. Lysate concentration was assessed using bicinchoninic acid assay (BCA).

### 2.4. Antibodies

The following antibodies were used in the study: anti-BAF155 (Abcam, Cambridge, UK, ab172638, IP and Western blotting), anti-BAF170 (Santa Cruz Biotechnology, Inc., Dallas, TX, USA sc166537, Western blotting), anti-BRD9 (Active Motif, Carlsbad, CA, USA 61537, IP and Western blotting), anti-BRD9 (Bethyl Laboratories, Inc., Montgomery, TX, USA, A303-781A, immunocytochemistry), anti-BRD9 (ThermoFisher, customized using porcine BRD9 peptide sequence, IP and Western blotting), anti-BRG1 (Abcam, ab110641, IP and Western blotting), anti-GLTSCR1 (Santa Cruz, sc51508, IP, Western blotting, and immunocytochemistry), anti-SNF5 (Abcam, ab88589, immunocytochemistry), mouse IgG (Abcam, ab6708, IP and Western blotting), rabbit IgG (Abcam, ab171870, IP and Western blotting), mouse IgGκ BP-HRP (Santa Cruz, sc516102, Western blotting), goat anti-rabbit IgG (H + L) (ThermoFisher (Invitrogen), A27036, Western blotting), goat anti-rabbit IgG-TRITC (Sigma, T6778, immunocytochemistry), and anti-rabbit IgG (whole molecule)-FITC (Sigma, F0382).

### 2.5. Immunoprecipitation

Nuclear lysates from cells were pre-cleared with mouse IgG-conjugated (Abcam) protein A/G magnetic beads (ThermoFisher) or rabbit IgG-conjugated (Abcam) protein A magnetic beads (ThermoFisher). One microgram of the respective IgG was used per 0.4 mg lysate for immunoprecipitation. Lysates were incubated with primary antibody and rotated overnight at 4 °C. Immunocomplexes were captured using either protein A/G or protein A magnetic beads following a 3-h incubation. The beads were washed 4 times in lysis buffer. Proteins were eluted in SDS-page loading dye (Boston Bioproducts, Ashland, MA, USA) containing 10% 2-mercaptoethanol at 70 °C for 10 min.

### 2.6. Immunoblot Analysis

Proteins were separated on a 10% SDS-polyacrylamide gel (Bio-Rad Laboratories, Hercules, CA, USA) and transferred to a nitrocellulose membrane (ThermoFisher). For detection using Clarity Max Western ECL Substrate (Bio-Rad), the membrane was blocked with 5% skim milk in TBS containing 0.1% Tween-20 (PBST) at room temperature for 1 h and then incubated with primary antibody overnight at 4 °C. Primary antibodies were detected on the membrane via incubation with HRP-conjugated goat anti-rabbit or anti-mouse secondary antibodies for 1 h at room temperature. The membrane signal was developed using Clarity Max (Bio-Rad). For detection using Clean-Blot IP Detection Kit (ThermoFisher), the membrane was blocked as previously described, incubated with primary antibody, and developed according to kit instructions. All membranes were visualized using (FluorChem R, ProteinSimple, San Jose, CA, USA).

### 2.7. Immunocytochemistry

All washes were carried out at 4 °C for 10 min each unless otherwise noted. Controls used in each cell type replicate set included secondary antibody-only incubation (to control for non-specific binding) and cells incubated with no antibodies (to control for background fluorescence). Fibroblast and trophectoderm cells were cultured on coverslips treated with Attachment Factor (ThermoFisher). The cells were washed 3 times in PBS and fixed in 7:3 methanol:acetone at −20 °C for 6 min, then washed three times in PBST. The cells were permeabilized in PBS with 1% TritonX-100 for 1 h at 4 °C then blocked overnight at 4 °C in blocking buffer (0.1 M glycine, 1% goat serum, 0.01 TritonX-100, 1% powdered nonfat dry milk, 0.5% BSA, 0.02% sodium azide in PBS). Cells were incubated overnight at 4 °C with primary antibody diluted in PBST, washed 3 times in PBST, then incubated overnight at 4 °C with goat anti-rabbit (ThermoFisher) or goat anti-mouse (Santa Cruz) secondary antibodies conjugated to TRITC or FITC, respectively. The cells were washed three times in PBST, incubated for 1 h with 2 μg/mL Hoechst 33342 diluted in PBS, and then washed once in PBST for 30 min. Coverslips were mounted onto slides with Vectashield mounting medium (Vector Laboratories, Inc., Burlingame, CA, USA) and sealed with acetone-free nail polish. 

Cleavage stage embryos were fixed at 4 °C for 1 h in 3.7% paraformaldehyde, then washed three times in PBST, each wash for 15 min. Blocking, permeabilization, antibody incubation, and imaging were performed as described above for trophectoderm and fibroblast cell lines. Controls used in each replicate of oocyte and embryonic immunocytochemical staining were performed as described above for each cell line. All samples were imaged using a Nikon A1R-MP multiphoton confocal microscope (Nikon Instruments Inc., Melville, NY, USA) using de-scanned detectors and laser lines at 561 nm (TRITC), 488 nm (FITC), and 408 nm (Hoechst). 

### 2.8. RNA-Seq Analysis

Raw read files were obtained from the NCBI Sequence Read Archive (SRA) from the following accessions: PRJNA601900, PRJNA686960, PRJNA580004, PRJNA778857, and PRJNA798047. Read quality determination and trimming were performed using Trim Galore [35] with the --fastqc option. Reads were aligned to the Sscrofa11.1 reference genome (GCF_000003025.6; obtained from https://www.ncbi.nlm.nih.gov/datasets/genome/GCF_000003025.6/ (accessed on 3 December 2023)) using HISAT2 [36]. Count files were generated using the htseq-count tool from HTSeq [37] with the mode option set to intersection-nonempty to handle reads overlapping >1 feature. Normalized and log2CPM transformed expression data were generated using Limma [38,39,40] with trimmed mean of M values (TMM) [41] normalization.

### 2.9. Statistical Analysis

Due to differences in RNA-seq methodologies, samples from oocytes (*n* = 5), 4-cell embryos (*n* = 5), and blastocysts (*n* = 6) were evaluated as one group, while samples from PTr2 cells (*n* = 3) and PFF cells (*n* = 3) were evaluated as a separate group. For evaluating oocyte, 4-cell embryo, and blastocyst expression data, the Kruskal-Wallis rank sum test was used to identify any statistically significant differences between sample groups. Pairwise comparisons of significant groups were performed using the Wilcoxon rank-sum exact test with Benjamini-Hochberg *p*-value adjustment. For evaluating PTr2 and PFF expression data, a Welch Two Sample *t*-test was performed. For all analyses, α = 0.05 with a 95% confidence interval was used. Fisher’s exact *t*-tests were performed using categorical data to calculate the *p* values from biological replicates in R for immunoblotting and immunochemical staining assays.

## 3. Results

### 3.1. GBAF Characteristic Subunits BRD9 and GLTSCR1 Are Present in Porcine Oocytes, Embryos, and Cell Lines

To determine the localization of GBAF subunits BRD9 and GLTSCR1 in porcine oocytes, embryos, and cell lines, immunocytochemistry was performed using commercial antibodies against the subunits of interest. All results presented here represent data collected from three independent replicates for each sample type. Samples were counterstained with Hoechst for nuclei identification. Non-specific staining was not detected above background levels in secondary or background controls performed for any of the analyzed samples. Positive controls staining for other BAF subcomplex subunits with known localization patterns were performed; to allow for dual FITC/TRITC staining, the host species of the target subunit antibody was paired with a positive control antibody raised in a different species. That is, BRD9 (rabbit) staining was done together with the BAF subunit SNF5/BAF47 (mouse) as a positive control, while GLTSCR1 (mouse) staining was done concurrently with BRG1 (rabbit).

**Figure 1:**
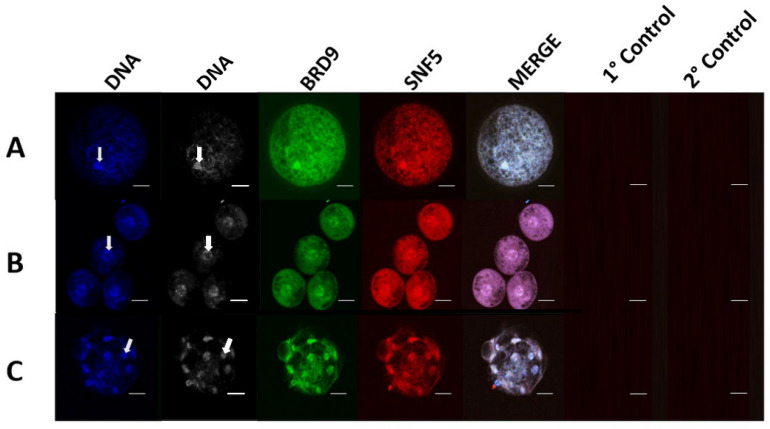
Immunocytochemical localization shows BRD9 in porcine oocytes and embryos produced in vitro. Panels (**A**), (**B**) and (**C**) contain representative images of a GV-stage oocyte, 4-cell stage embryo, and blastocyst-stage embryo, respectively. For each panel, from left to right, DNA staining is shown in blue, BRD9 intracellular localization is shown in green, SNF5 intracellular localization is shown in red, followed by a merged channel image. Additionally, primary controls (no primary antibody) and secondary controls (no secondary antibody) collected with each sample type are displayed. Arrows indicate the location of a representative nucleus for each developmental stage.

**Figure 2:**
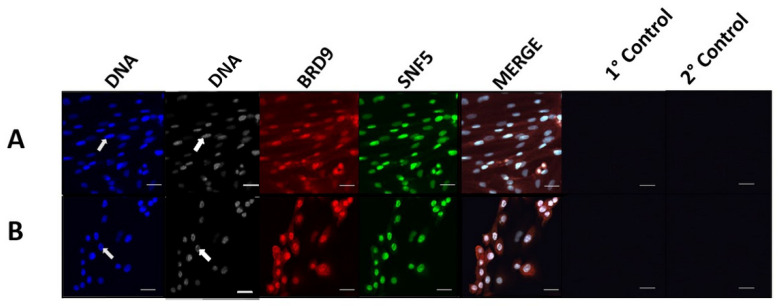
Immunocytochemical localization shows BRD9 in porcine cell lines. Panels (**A**) and (**B**) contain representative images of porcine fetal fibroblast and porcine trophectoderm cells, respectively. For both panels, from left to right, DNA staining is shown in blue, SNF5 intracellular localization is shown in green, and BRD9 intracellular localization is shown in red, followed by a merged channel image. Primary controls (no primary antibody) and secondary controls (no secondary antibody) collected with each cell type are displayed. Arrows indicate the location of a representative nucleus for each developmental stage.

In germinal vesicle-stage oocytes (*n* = 30), 4-cell stage embryos (*n* = 28), and blastocyst-stage embryos (*n* = 27), BRD9 displayed both nuclear and cytoplasmic localization, though nuclear enrichment was distinguishable amid the cytoplasmic staining patterns (Figure 1A–C). Punctate regions of BRD9 nuclear signal were seen in 4-cell stage embryos (Figure 1B) Cytoplasmic signal of BRD9 appeared to be weaker in blastocyst-stage embryos compared to earlier-stage embryos and oocytes (Figure 1C). In fetal fibroblasts and trophectoderm cells, strong nuclear enrichment of BRD9 was observed (Figure 2A,B). Cytoplasmic BRD9 signal was also discernible across all replicates.

**Figure 3:**
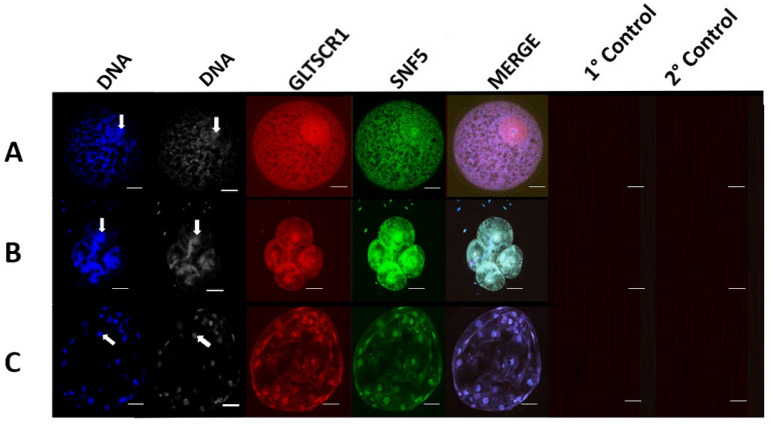
Immunocytochemical localization shows GLTSCR1 in porcine oocytes and embryos produced in vitro. Panels (**A**), (**B**) and (**C**) contain representative images of a GV-stage oocyte, 4-cell stage embryo, and blastocyst-stage embryo, respectively. For each panel, from left to right, DNA staining is shown in blue, SNF5 intracellular localization is shown in green, GLTSCR1 intracellular localization is shown in red, followed by a merged channel image. Primary controls (no primary antibody) and secondary controls (no secondary antibody) collected with each sample type are displayed. Arrows indicate the location of a representative nucleus for each developmental stage.

**Figure 4:**
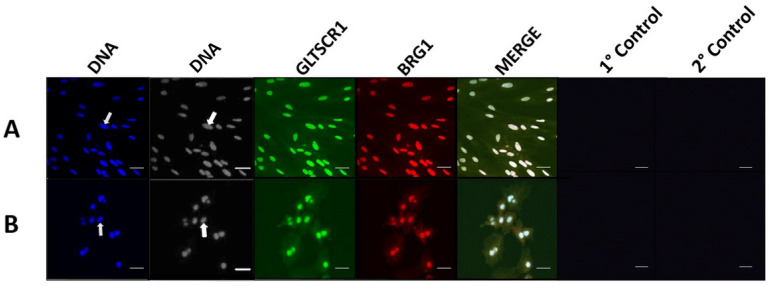
Immunocytochemical localization shows GLTSCR1 in porcine cell lines. Panels (**A**) and (**B**) contain representative images of porcine fetal fibroblast and porcine trophectoderm cells, respectively. For both panels, from left to right, DNA staining is shown in blue, GLTSCR1 intracellular localization is shown in green, BRG1 intracellular localization is shown in red, followed by a merged channel image. Primary controls (no primary antibody) and secondary controls (no secondary antibody) collected with each cell type are displayed. Arrows indicate the location of a representative nucleus for each developmental stage.

GLTSCR1 signal displayed nuclear and cytoplasmic ubiquity in germinal vesicle-stage oocytes (*n* = 27) and 4-cell stage embryos (*n* = 24; Figure 3A,B). GLTSCR1 was detected predominantly in nuclear regions of blastocyst-stage embryos, though weak cytoplasmic association was also present (*n* = 30; Figure 3C). In fibroblast and trophectoderm cells, GLTSCR1 adopted clear nuclear localization (Figure 4A,B). The weak cytoplasmic signal was observed in both cell lines, though cytoplasmic enrichment of GLTSCR1 was slightly stronger in trophectoderm samples (Figure 4B). Regarding the presence of BRD9 and GLTSCR1 across all sample types, no significant difference in subunit-specific signal prevalence was identified (Fisher’s exact t; *p* = 1). 

These data indicate that GBAF is present during early embryonic development in swine, though further research is needed to determine mechanistic details concerning whether GBAF may play a role in directing the progression of key developmental stages prior to implantation and maternal recognition of pregnancy.

### 3.2. GLTSCR1 Is Present in Porcine Pre-Implantation and Early Development Cell Lines and Interacts with BAF170

To explore intra-complex subunit interactions within GBAF, co-immunoprecipitation was performed with BRD9, GLTSCR1, and other BAF subunits. Weak interaction between GLTSCR1 and BRG1 was observed in both fetal fibroblast and trophectoderm cells (Figure 5A–C; Supplementary Figures S1–S12). 

**Figure 5:**
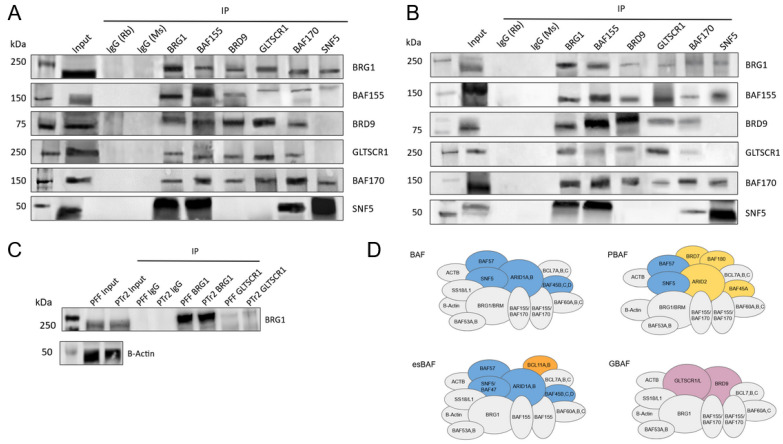
Detection of BRD9 and GLTSCR1 in porcine cell lines shows a GLTSCR1/BAF170 interaction. Panels (**A**) and (**B**) show co-immunoprecipitation results in porcine fetal fibroblast and trophectoderm cells, respectively. For each panel, from left to right, GBAF-associated subunit immunoprecipitation was performed with rabbit and mouse IgG, BRG1, BAF155, BRD9, GLTSCR1, BAF170, and BAF47 (SNF5). Co-immunoprecipitation analysis for each GBAF-associated subunit was performed with BRG1, BAF155, BRD9, GLTSCR1, BAF170, and BAF47. Panel (**C**) shows a relative signal intensity comparison between BRG1 and GLTSCR1 in fetal fibroblast (PFF) and trophectoderm (TR2) cells. Panel (**D**) is a schematic depiction of select BAF-family subcomplexes and their constituent subunits. Subunits shared between complexes are colored gray, subunits found in some BAF subcomplexes but not GBAF are colored blue, PBAF-specific subunits are in yellow, an esBAF-specific subunit is in orange, and GBAF-specific subunits are in purple.

To provide a more direct comparison of BRG1 and GLTSCR1 signal between cell lines, co-immunoprecipitation between the two subunits was performed on the same blot (Figure 5C). Consistent with results seen in earlier panels, weak GLTSCR1 association with BRG1 was observed in both cell types, indicating that GLTSCR1 maintains a much lower relative abundance across these samples when compared to other GBAF subunits. Notably, an interaction between GLTSCR1 and BAF170 was observed via reciprocal co-immunoprecipitation in extracts derived from both fibroblast and trophectoderm cells, pointing to a previously undescribed interaction between these two subunits (Figure 5A,B,D).

### 3.3. GBAF Subunit Expression Varies across Developmental Stages

**Figure 6:**
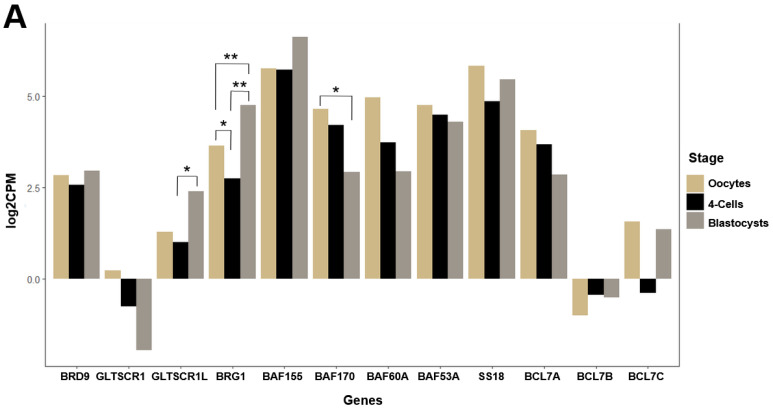

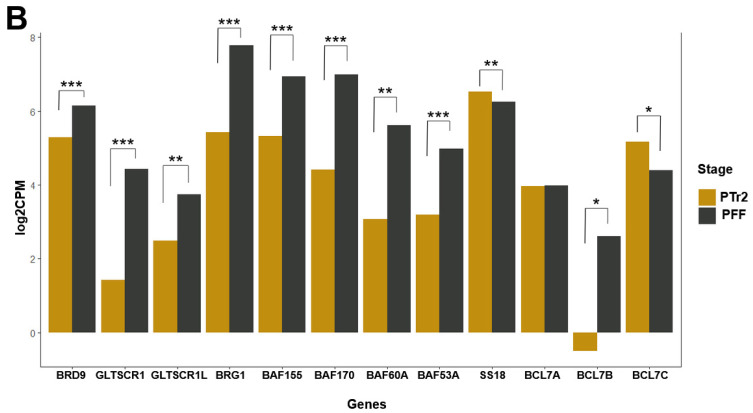
Transcriptional dynamics of GBAF subunits across different early developmental stages. For each GBAF subunit, the average TMM-normalized log2-transformed CPM value pertaining to the representative developmental stage is given. Panel (**A**) depicts findings in oocytes, 4-cell embryos, and blastocysts, while Panel (**B**) depicts findings for PTr2 and PFF cells. For subunits demonstrating statistically significant differences in expression levels between developmental stages, significance level symbols were assigned according to the following *p*-value ranges: <0.05 (*), <0.01 (**), <0.001 (***).

BRD9 displayed generally comparable expression levels in porcine oocytes, 4-cell embryos, and blastocysts, and no statistically significant differences were seen across these stages (*p* = 0.86, Χ^2^ = 0.29, df = 2) (Figure 6A; Table 1). Expression levels of GLTSCR1 remained consistently low in samples across all three pre-implantation stages (*p* = 0.270, Χ^2^ = 2.43, df = 2), contrasting with those of its mutually exclusive paralog, GLTSCR1L (*p* = 0.044, Χ^2^ = 6.23, df = 2) (Figure 6A; Table 1). Of the developmental stages represented in this comparison, 4-cell embryos and blastocysts had significantly different (*p*_adj_ = 0.026) levels of GLTSCR1L expression (Figure 6A; Table 1). Relative to GBAF-specific subunits, subunits found in GBAF and other BAF family subcomplexes consistently exhibited higher average expression levels across the represented developmental stages. Of these subunits, BAF170 expression between oocytes and blastocysts was significantly different (*p* = 0.018, Χ^2^ = 7.99, df = 2; *p*_adj_ = 0.026), and BRG1 had significantly different (*p* = 0.002, Χ^2^ = 12.53, df = 2) expression across all developmental stage comparisons, with oocytes and 4-cell embryos (*p*_adj_ = 0.0317), oocytes and blastocysts (*p*_adj_ = 0.0065), and 4-cell embryos and blastocysts (*p*_adj_ = 0.0065) (Figure 6A; Table 1).

In PTr2 and PFF cells, the average expression of GBAF subunits was consistently significantly higher in PFF cells relative to PTr2 cells, as demonstrated by BRD9 (*p* = 2.168 × 10^−6^, t = −52.74), GLTSCR1 (*p* = 1.621 × 10^−4^, t = −45.61), and GLTSCR1L (*p* = 2.000 × 10^−2^, t = −6.47) (Figure 6B; Table 2). Indeed, a clear difference was seen in GLTSCR1 expression between the cell types, with the mean expression level of this subunit in PFF cells more than double that of PTr2 cells. In addition to this, several other BAF family subunits exhibited higher relative expression levels in PFF cells compared to PTr2 cells, with BRG1 (*p* = 4.575 × 10^−4^, t = −45.80)), BAF155 (*p* = 4.609 × 10^−6^, t = −39.99), BAF170 (2.071 × 10^−4^, t = −50.17), BAF53A (*p* = 1.425 × 10^−5^, t = −27.77), BAF60A (*p* = 0.001, t = −21.19), and BCL7B (*p* = 0.024, t = −6.19) representing the subunits with significantly higher mean expression levels in PFF cells (Figure 6B; Table 2). Two subunits, SS18 (*p* = 0.002, t = 7.52) and BCL7C (*p* = 0.017, t = 6.27) had significantly higher means in PTr2 cells, while no difference in mean BCL7A expression was observed between the two cell types (Figure 6B; Table 2).

## 4. Discussion

There is no shortage of findings supporting the implications of epigenetic regulation of livestock performance and human health, though, in the case of large animal studies, the majority of this research has focused on evaluating changes in global genomic methylation patterns following exposure to specific environmental conditions [42]. In cattle, for example, aberrant DNA methylation patterns are associated with suboptimal sperm function and development [43,44], as well as the ability of the bovine immune system to clear infections of certain pathogens, such as *Mycobacterium bovis* [45]. Additionally, DNA methylation can impact heat stress tolerance in swine [46,47] and sheep [48,49]. Recent research has also determined that vaccination against the porcine reproductive and respiratory syndrome virus (PRRSV) may result in heritable, epigenetic changes that could potentially be exploited as a way to develop PRRSV-resistant animals [50]. While these studies have accentuated the significance of epigenetic regulation in livestock performance and human health, the narrative involving chromatin remodeling complexes is rapidly evolving, particularly concerning abnormal subunit expression in BAF subcomplexes and their implications in developmental dysfunction. This study identified the GBAF chromatin remodeling complex and examined the transcriptional dynamics of its subunits across various porcine developmental stages and cell lines, contributing to the growing understanding of epigenetic regulation within both swine and mammalian development. 

As mentioned previously, abnormal expression of BAF subcomplex subunits has been associated with developmental dysfunction in animals. In swine, BRG1, a BAF complex central ATPase, plays a crucial role in zygotic genome activation [28], and the BAF-associated subunit ARID1A (for AT-rich interaction domain 1A) modulates embryogenesis through the blastocyst stage [31]. Mouse models further emphasize the requisite roles of specific BAF subunits in regulating gene expression within embryonic stem cells [26] and during germ-layer formation [27], highlighting the intimate link between chromatin remodelers and embryonic development. The value of investigating the intersectionality between early mammalian development and epigenetic influence on progeny health and performance outcomes is further underscored by the expanding body of evidence supporting that prenatal exposure to different stressors can influence long-term phenotypic changes in offspring [51,52,53,54,55]. In addition to developmental implications, disease-associated mutations across several BAF subunits further underscore the importance of continued investigation into the presence and compositional requirements of these subcomplexes. Research by Michel et al. demonstrated that loss of core BAF subunit functionality in BAF-perturbed cancers (e.g., BAF47 perturbation in MRT and SS) leads to a dependency of these cancer types on GBAF for gene expression maintenance, a relationship which can be compromised via BRD9 depletion [18]. These findings demonstrate that chromatin remodeler control of gene expression has broad implications for both mammalian development and disease, emphasizing the potential role of GBAF as an important epigenetic modulator. 

The results presented here indicate that BRD9 and GLTSCR1, and GBAF by extension, are ubiquitously expressed in both pre- and post-implantation stages of swine embryonic and extraembryonic development. This is consistent with the previous identification of other BAF subcomplex subunits BAF155 and BAF53A during peri-implantation time points [30], both of which have been identified as GBAF constituents [11]. While BRD9 and GLTSCR1 have been shown to modulate mouse ESC survival [11] and naïve pluripotency [14], respectively, to our knowledge, the expression of these GBAF subunits have not been described in swine prior to this work. 

Notably, our results suggest a novel association between GLTSCR1 and BAF170 in porcine cells, hinting at a potential BAF155/BAF170 heterodimer in porcine GBAF. BAF155 and BAF170 serve as scaffolding subunits in SWI/SNF subcomplexes, and both are expressed in porcine cleavage stage embryos [30]. BAF155 and BAF170 can exist as homodimers or heterodimers depending upon the subcomplex and species [9,12,56]. For example, in murine esBAF, a BAF155 homodimer exists [12], while a BAF155/170 heterodimer is detectable in human esBAF [57]. Furthermore, existing literature reports GBAF compositions within murine and human cell lines which include a BAF155 homodimer [11,14]. However, to date, a GBAF composition containing BAF170 has not been reported in any species. Our results suggest a novel association between GLTSCR1 and BAF170 in porcine trophectoderm and fetal cells, and these data may imply the existence of a previously undescribed, species-specific BAF155/BAF170 heterodimer in porcine GBAF. The implications of this subunit’s presence in porcine GBAF are yet unknown, and future investigation may be warranted to determine the mechanistic contributions of BAF170 as a GBAF constituent. 

Our study also profiles GBAF subunit expression across swine preimplantation stages, trophectoderm, and fetal fibroblast cell lines. The expression levels of GBAF-specific subunits BRD9, GLTSCR1, and GLTSCR1L were comparatively lower than that of subunits also shared with other BAF family subcomplexes, and this may be attributable to a relatively lower abundance of GBAF during the represented developmental stages compared to other BAF family subcomplexes. GLTSCR1 and GLTSCR1L expression levels were particularly low across all three pre-implantation development stages and in PTr2 cells. While BRD9 expression levels were also low in comparison to some other BAF family subunits, its expression levels were higher than those of GLTSCR1 and GLTSCR1L. Subunits of hetero-oligomeric chromatin remodeling complexes may be expected to be expressed at similar levels, but numerous examples exist of multi-subunit protein complexes with stoichiometries that do not necessarily match with the actual expression levels of their constituent subunits [58]. In the case of the differences in expression levels seen between BRD9 and GLTSCR1/*L* in the represented developmental stages, BRD9 may have other functionalities beyond its contributions as part of GBAF. Future work, dependent upon the availability of antibodies compatible with swine samples, may endeavor to explore the functional contribution(s) of BRD9 during early swine development and determine whether this bromodomain-containing protein plays a necessary role in developmental contexts. Furthermore, the overall dearth of RNA-sequencing data from samples representing these early developmental stages in swine would also aid in increasing statistical power and improve the strength of conclusions as to whether these findings are accurate population-wide. 

Within oocytes, SWI/SNF chromatin remodeling complex subunits are known to regulate critical functions such as meiotic progression [59], and disruptions to this process may compromise oocyte viability. The 4-cell embryo stage in pigs (and humans) is the point of zygotic genome activation (ZGA), after which the embryo ceases to utilize maternal mRNA resources and must synthesize its mRNA for protein production. It has been shown that, around the time of ZGA, chromatin is in a broadly permissible state [60] and gene expression is promoted, in part, by a coordinated regulatory system involving maternal TIFα (for transcription intermediary factor), histone acetyltransferases, and SWI/SNF chromatin remodeling complexes [61]. In the blastocyst stage, as has been mentioned previously, numerous SWI/SNF subcomplex subunits are necessary for peri-implantation developmental progression and embryonic viability (reviewed by [62]). Less is known about how chromatin remodeling complexes contribute to trophoblast and fibroblast identity and function, particularly in swine, though the breadth of transcriptional control accomplished by different remodelers throughout early development and beyond would suggest that these epigenetic mechanisms likely still have some level of contribution within these cell types. Cumulatively, the ubiquitous presence of GBAF subunits across these developmental stages, even at relatively lower levels of expression compared to other BAF subunits, suggests that this chromatin remodeling complex may play a role in regulating chromatin architecture during early swine development. However, its exact mechanistic contributions in these contexts remain to be determined. 

## 5. Conclusions

Taken together, results reported here document the existence of GBAF in porcine early embryonic, extraembryonic, and fetal developmental stages, and the transcriptional dynamics of GBAF subunits unveiled in this study contribute to the evolving narrative of chromatin remodeling complex involvement in mammalian development. The identified subunit association and transcriptional details underscore the intricate interplay between epigenetic regulation and developmental processes, providing valuable insights for future research into the potential implications of GBAF as a modulator of key mammalian developmental events in both pre-and post-implantation contexts and its potential epigenetic regulatory contributions to both human and livestock health. 

## Figures and Tables

**Table 1:** Statistical summary of Kruskal-Wallis Rank Sum Test and Wilcoxon Rank Sum Exact test for comparing expression means between oocytes, 4-cell embryos, and blastocysts.

			Kruskal-Wallis Rank-Sum Test			Wilcoxon Rank-Sum Exact Test	
Subunit	Mean	SD	Χ^2^	df	*p*-Value	Comparison	*p*_adj_
BRD9							
Oocytes	2.84	1.47	0.29	2	0.863	Oocytes—4-cells	0.93
4-Cell embryos	2.56	1.69				Oocytes—blastocysts	0.93
Blastocysts	2.96	1.70				4-cells-blastocysts	0.93
GLTSCR1							
Oocytes	0.23	1.82	2.43	2	0.297	Oocytes—4-cells	0.420
4-Cell embryos	−0.76	1.32				Oocytes—blastocysts	0.420
Blastocysts	−1.96	2.23				4-cells-blastocysts	0.420
GLTSCR1L							
Oocytes	1.29	1.39	6.28	2	0.044	Oocytes—4-cells	0.690
4-Cell embryos	0.99	0.62				Oocytes—blastocysts	0.266
Blastocysts	2.40	0.80				4-cells-blastocysts	0.026
BRG1							
Oocytes	3.64	0.21	12.53	2	0.002	Oocytes—4-cells	0.032
4-Cell embryos	2.74	0.77				Oocytes—blastocysts	0.007
Blastocysts	4.76	0.95				4-cells-blastocysts	0.007
BAF155							
Oocytes	5.76	0.50	5.09	2	0.079	Oocytes—4-cells	0.690
4-Cell embryos	5.73	1.46				Oocytes—blastocysts	0.013
Blastocysts	6.62	0.22				4-cells-blastocysts	0.690
BAF170							
Oocytes	4.64	0.45	7.99	2	0.018	Oocytes—4-cells	0.421
4-Cell embryos	4.21	0.84				Oocytes—blastocysts	0.026
Blastocysts	2.92	0.76				4-cells-blastocysts	0.078
BAF53A							
Oocytes	4.75	0.37	2.31	2	0.315	Oocytes—4-cells	0.840
4-Cell embryos	4.49	1.51				Oocytes—blastocysts	0.490
Blastocysts	4.30	0.56				4-cells-blastocysts	0.490
BAF60A							
Oocytes	4.97	1.05	5.76	2	0.056	Oocytes—4-cells	0.032
4-Cell embryos	3.72	1.78				Oocytes—blastocysts	0.007
Blastocysts	2.94	0.38				4-cells-blastocysts	0.007
SS18							
Oocytes	5.82	0.97	1.32	2	0.516	Oocytes—4-cells	0.790
4-Cell embryos	4.86	1.31				Oocytes—blastocysts	0.790
Blastocysts	5.46	1.23				4-cells-blastocysts	0.790
BCL7A							
Oocytes	4.06	1.33	1.58	2	0.453	Oocytes—4-cells	0.840
4-Cell embryos	3.68	1.02				Oocytes—blastocysts	0.740
Blastocysts	2.85	1.54				4-cells-blastocysts	0.810
BCL7B							
Oocytes	−1.02	1.84	0.26	2	0.877	Oocytes—4-cells	0.930
4-Cell embryos	−0.45	2.35				Oocytes—blastocysts	0.930
Blastocysts	−0.51	1.64				4-cells-blastocysts	0.930
BCL7C							
Oocytes	1.57	1.15	3.51	2	0.173	Oocytes—4-cells	0.330
4-Cell embryos	−0.39	2.08				Oocytes—blastocysts	0.790
Blastocysts	1.35	0.80				4-cells-blastocysts	0.250

**Table 2:** Statistical summary of Welch’s *t*-test results for comparing expression means between trophoblast (PTr2) and fetal fibroblast (PFF) cells.

Comparison (PTr2-PFF)	Mean_PTr2_	SD_PTr2_	Mean_PFF_	SD_PFF_	t	df	95% Confidence Interval		*p*-Value
							Lower	Upper	
BRD9	5.29	0.02	6.15	0.02	−52.74	3.65	−0.91	−0.81	2.168 × 10^−6^
GLTSCR1	1.41	0.03	4.42	0.11	−45.61	2.35	−3.25	−2.76	1.621 × 10^−4^
GLTSCR1L	2.49	0.33	3.74	0.06	−6.47	2.12	−2.04	−0.46	0.02
BRG1	5.42	0.09	7.78	0.01	−45.80	2.01	−2.59	−2.14	4.575 × 10^−4^
BAF155	5.32	0.06	6.93	0.04	−39.99	3.74	−1.73	−1.50	4.609 × 10^−6^
BAF170	4.40	0.09	6.98	0.02	−50.17	2.20	−2.78	−2.38	2.017 × 10^−4^
BAF60A	2.08	0.20	5.61	0.05	−21.21	2.21	−3.01	−2.06	0.001
BAF53A	3.18	0.07	4.97	0.09	−27.77	3.84	−1.97	−1.61	1.425 × 10^−5^
SS18	6.53	0.04	6.24	0.05	7.52	3.98	0.18	0.38	0.002
BCL7A	3.96	0.08	3.99	0.06	−0.44	3.63	−0.20	0.15	0.682
BCL7B	−0.52	0.87	2.61	0.08	−6.19	2.04	−5.26	−0.99	0.024
BCL7C	5.16	0.06	4.40	0.20	6.27	2.31	0.30	1.23	0.017

## Data Availability

Sequencing files for oocytes, 4-cell embryos, and blastocysts were obtained from the Gene Expression Omnibus (GEO) repository for accessions GSE143848, GSE163620, and GSE139512. PFF and PTr2 sequencing reads were obtained from NCBI Sequence Read Archive (SRA) accessions PRJNA798047 and PRJNA778857, respectively. All other data generated or analyzed during this study are included in this published article (and its Supplementary Information Files).

## Supplementary Materials

The following supporting information can be downloaded at: https://www.mdpi.com/article/10.3390/ani14050773/s1, Figure S1: GBAF subunit signal is co-enriched with BRG1 in PFF cells. (A) Co-IP of (starting in lane 5) BRG1, BAF155, BRD9, GLTSCR1, BAF170, and SNF5 with BRG1 primary antibody. (B) Accompanying relative signal density plot for BRG1 primary blot. Figure S2: GBAF subunit signal is co-enriched with BAF155 in PFF cells. (A) Co-IP of (starting in lane 5) BRG1, BAF155, BRD9, GLTSCR1, BAF170, and SNF5 with BRG1 primary antibody. (B) Accompanying relative signal density plot for BAF155 primary blot. Figure S3: GBAF subunit BRD9 is detectable in PFF cells. (A) Co-IP of (starting in lane 5) BRG1, BAF155, BRD9, GLTSCR1, BAF170, and SNF5 with BRG1 primary antibody. (B) Accompanying relative signal density plot for BRD9 primary blot. Figure S4: GBAF subunit GLTSCR1 is detectable in PFF cells. (A) Co-IP of (starting in lane 5) BRG1, BAF155, BRD9, GLTSCR1, BAF170, and SNF5 with BRG1 primary antibody. (B) Accompanying relative signal density plot for GLTSCR1 primary blot. Figure S5: GBAF subunits BRD9 and GLTSCR1 are co-enriched with BAF170 in PFF cells. (A) Co-IP of (starting in lane 5) BRG1, BAF155, BRD9, GLTSCR1, BAF170, and SNF5 with BRG1 primary antibody. (B) Accompanying relative signal density plot for BAF170 primary blot. Figure S6: GBAF subunits BRD9 and GLTSCR1 are not coenriched with SNF5 in PFF cells. (A) Co-IP of (starting in lane 5) BRG1, BAF155, BRD9, GLTSCR1, BAF170, and SNF5 with BRG1 primary antibody. (B) Accompanying relative signal density plot for SNF5 primary blot. Figure S7: GBAF subunit signal is co-enriched with BRG1 in PTr2 cells. (A) Co-IP of (starting in lane 5) BRG1, BAF155, BRD9, GLTSCR1, BAF170, and SNF5 with BRG1 primary antibody. (B) Accompanying relative signal density plot for BRG1 primary blot. Figure S8: GBAF subunit signal is co-enriched with BAF155 in PTr2 cells. (A) Co-IP of (starting in lane 5) BRG1, BAF155, BRD9, GLTSCR1, BAF170, and SNF5 with BRG1 primary antibody. (B) Accompanying relative signal density plot for BAF155 primary blot. Figure S9: GBAF subunit BRD9 is detectable in PTr2 cells. (A) Co-IP of (starting in lane 5) BRG1, BAF155, BRD9, GLTSCR1, BAF170, and SNF5 with BRG1 primary antibody. (B) Accompanying relative signal density plot for BRD9 primary blot. Figure S10: GBAF subunit GLTSCR1 is detectable in PTr2 cells. (A) Co-IP of (starting in lane 5) BRG1, BAF155, BRD9, GLTSCR1, BAF170, and SNF5 with BRG1 primary antibody. (B) Accompanying relative signal density plot for GLTSCR1 primary blot. Figure S11: GBAF subunits BRD9 and GLTSCR1 are co-enriched with BAF170 in PTr2 cells. (A) Co-IP of (starting in lane 5) BRG1, BAF155, BRD9, GLTSCR1, BAF170, and SNF5 with BRG1 primary antibody. (B) Accompanying relative signal density plot for BAF170 primary blot. Figure S12: GBAF subunits BRD9 and GLTSCR1 are not co-enriched with SNF5 in PTr2 cells. (A) Co-IP of (starting in lane 5) BRG1, BAF155, BRD9, GLTSCR1, BAF170, and SNF5 with BRG1 primary antibody. (B) Accompanying relative signal density plot for SNF5 primary blot.
