# Supplementary material for: Identification of SWI/SNF Subcomplex GBAF Presence, Intra-Complex Interactions, and Transcriptional Dynamics during Early Porcine Development
Source: Animals (Basel). 2024 Feb 29;14(5):773. doi: 10.3390/ani14050773 (PMC10930984; doi:10.3390/ani14050773)
Supplement: Supplementary file 1 [file animals-14-00773-s001.zip › animals-2888806-supplementary.pdf]

Supplementary Materials

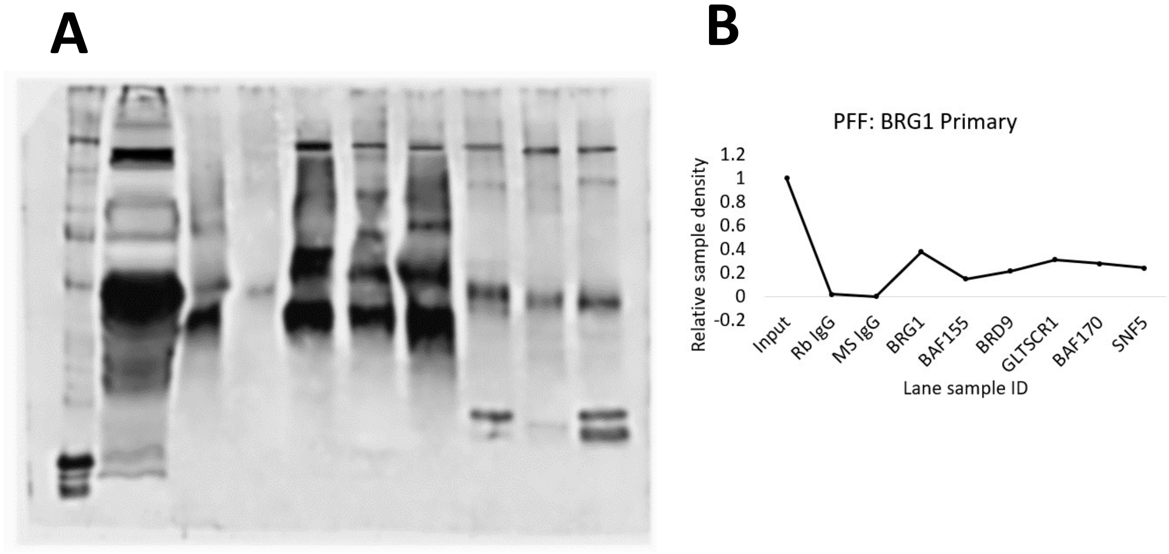

Figure S1. GBAF subunit signal is co-enriched with BRG1 in PFF cells. (**A**) Co-IP of (starting in lane 5) BRG1, BAF155, BRD9, GLTSCR1, BAF170, and SNF5 with BRG1 primary antibody. (**B**) Accompanying relative signal density plot for BRG1 primary blot.

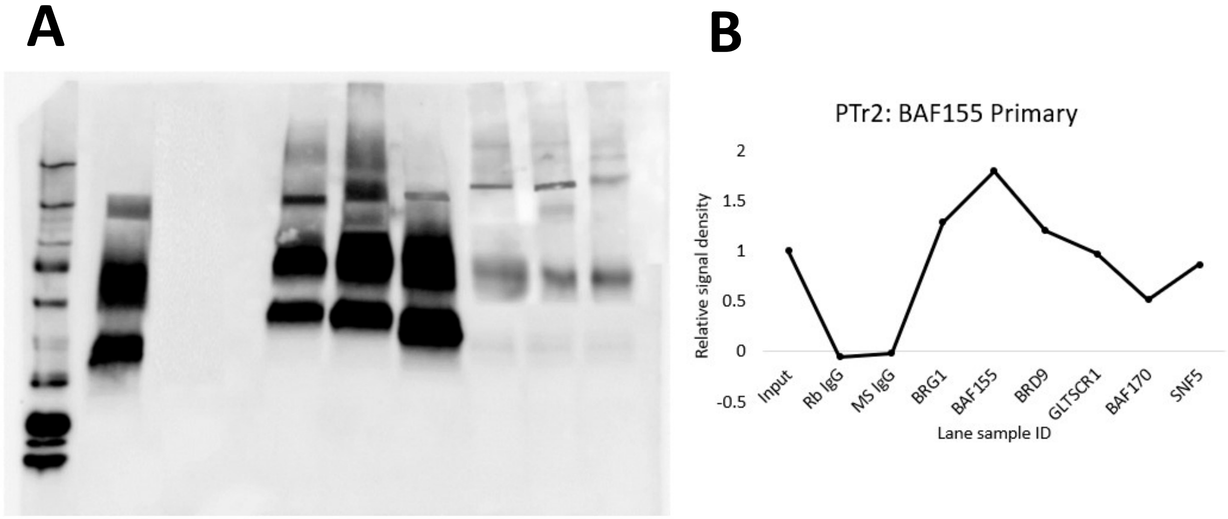

Figure S2. GBAF subunit signal is co-enriched with BAF155 in PFF cells. (**A**) Co-IP of (starting in lane 5) BRG1, BAF155, BRD9, GLTSCR1, BAF170, and SNF5 with BRG1 primary antibody. (**B**) Accompanying relative signal density plot for BAF155 primary blot.

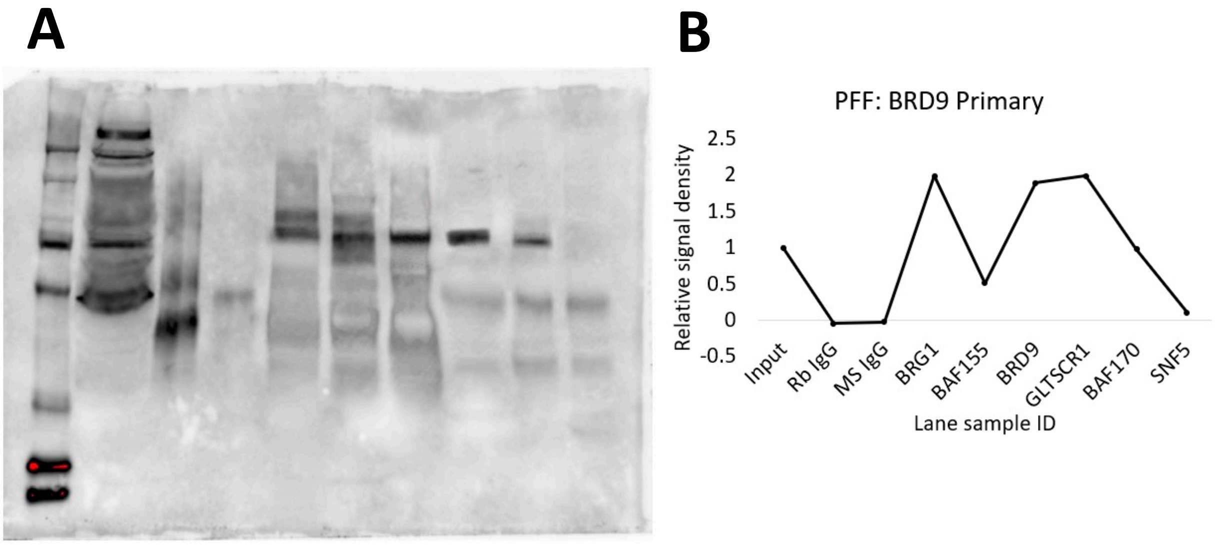

Figure S3. GBAF subunit BRD9 is detectable in PFF cells. (**A**) Co-IP of (starting in lane 5) BRG1, BAF155, BRD9, GLTSCR1, BAF170, and SNF5 with BRG1 primary antibody. (**B**) Accompanying relative signal density plot for BRD9 primary blot.

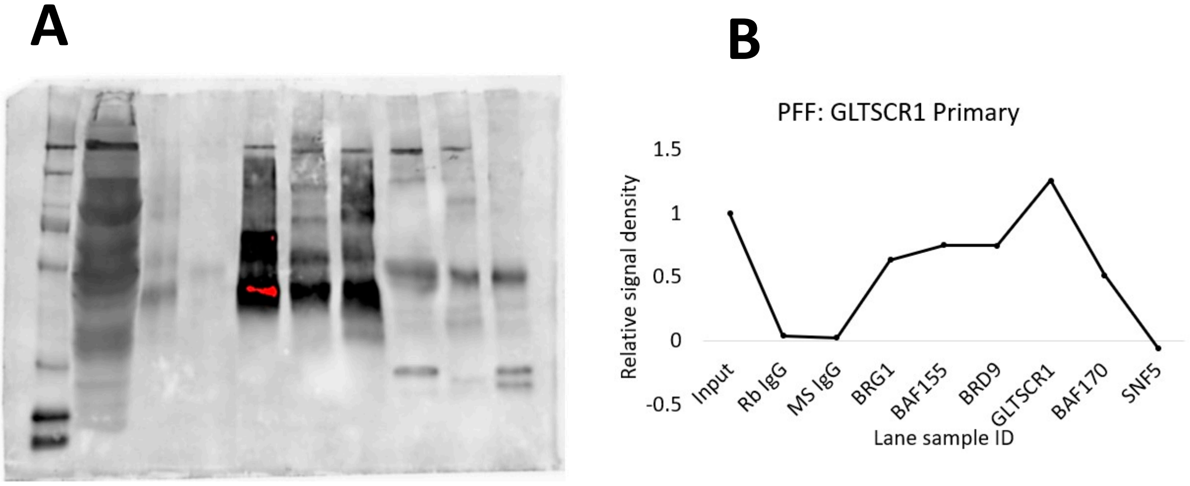

Figure S4. GBAF subunit GLTSCR1 is detectable in PFF cells. (**A**) Co-IP of (starting in lane 5) BRG1, BAF155, BRD9, GLTSCR1, BAF170, and SNF5 with BRG1 primary antibody. (**B**) Accompanying relative signal density plot for GLTSCR1 primary blot.

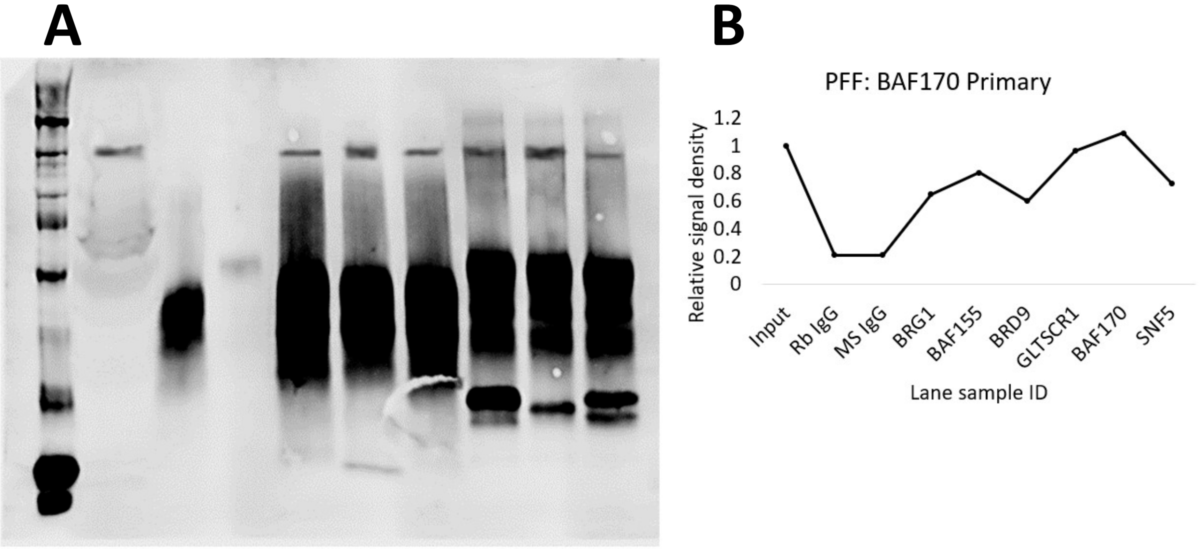

Figure S5. GBAF subunits BRD9 and GLTSCR1 are co-enriched with BAF170 in PFF cells. (**A**) Co-IP of (starting in lane 5) BRG1, BAF155, BRD9, GLTSCR1, BAF170, and SNF5 with BRG1 primary antibody. (**B**) Accompanying relative signal density plot for BAF170 primary blot.

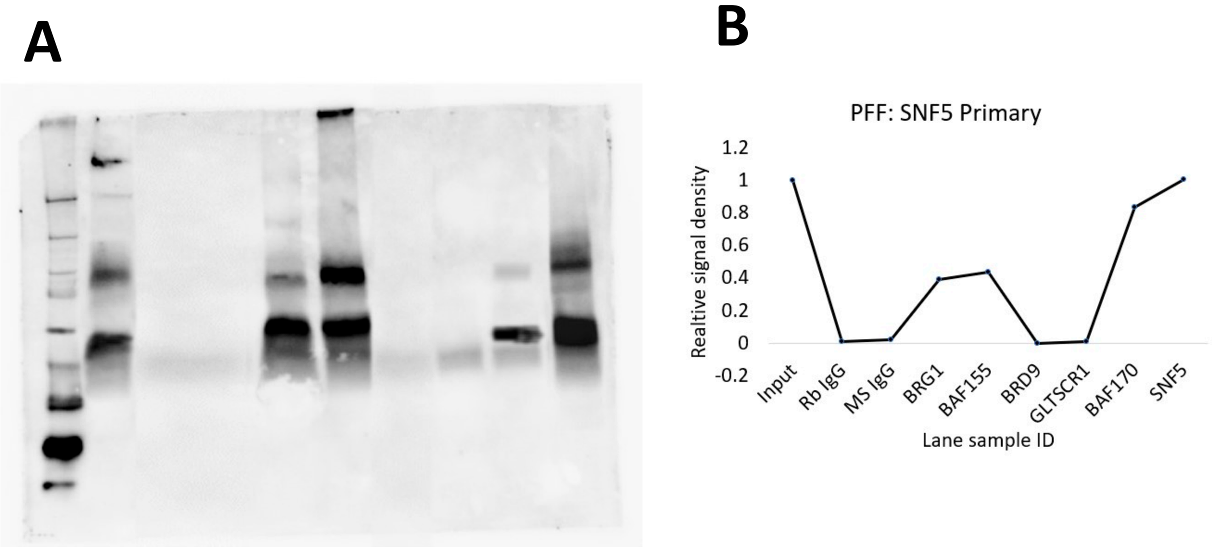

Figure S6. GBAF subunits BRD9 and GLTSCR1 are not co-enriched with SNF5 in PFF cells. (**A**) Co-IP of (starting in lane 5) BRG1, BAF155, BRD9, GLTSCR1, BAF170, and SNF5 with BRG1 primary antibody. (**B**) Accompanying relative signal density plot for SNF5 primary blot.

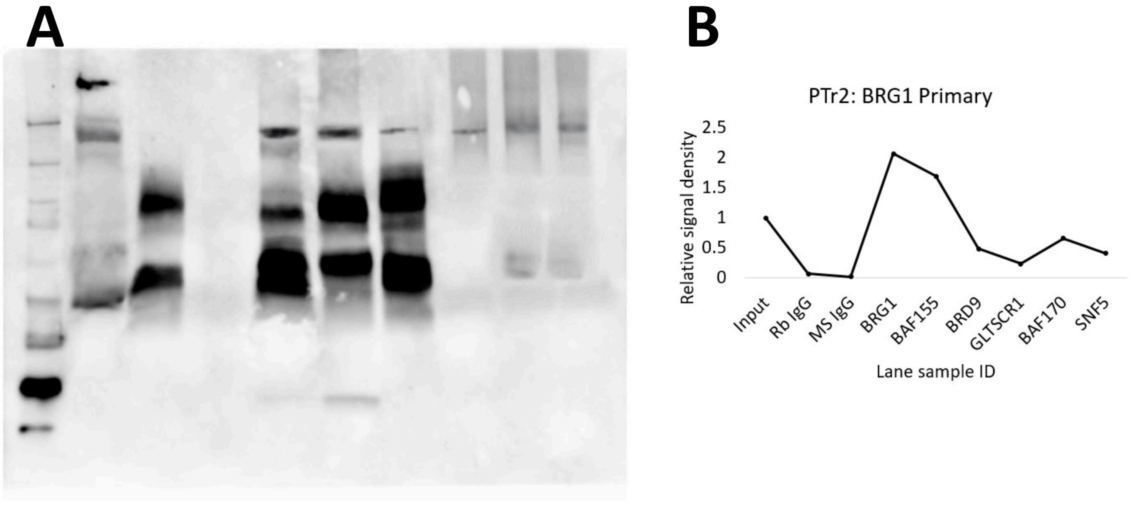

Figure S7. GBAF subunit signal is co-enriched with BRG1 in PTr2 cells. (**A**) Co-IP of (starting in lane 5) BRG1, BAF155, BRD9, GLTSCR1, BAF170, and SNF5 with BRG1 primary antibody. (**B**) Accompanying relative signal density plot for BRG1 primary blot.

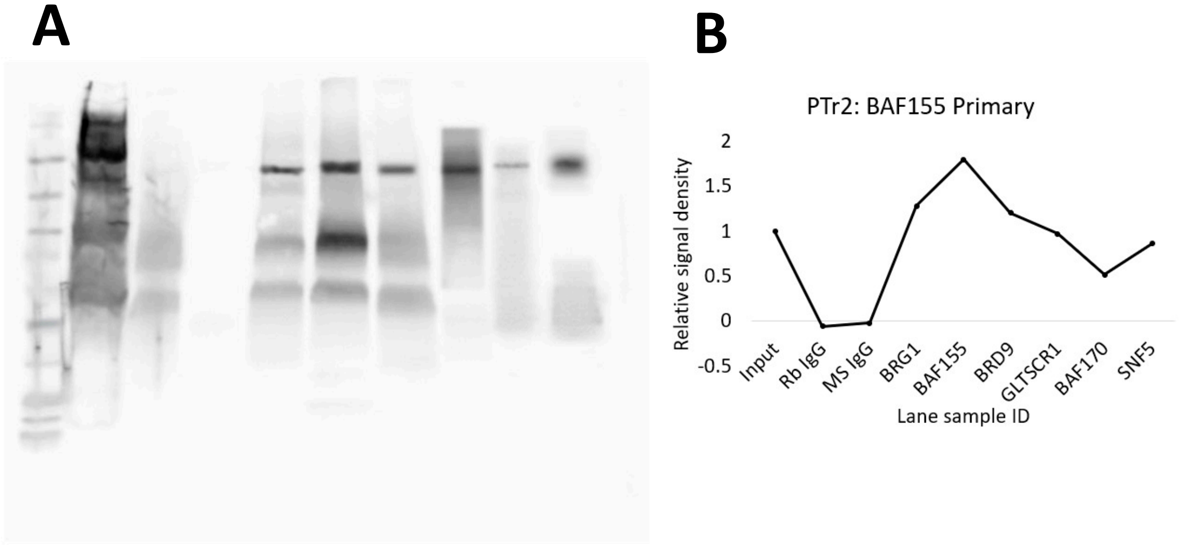

Figure S8. GBAF subunit signal is co-enriched with BAF155 in PTr2 cells. (**A**) Co-IP of (starting in lane 5) BRG1, BAF155, BRD9, GLTSCR1, BAF170, and SNF5 with BRG1 primary antibody. (**B**) Accompanying relative signal density plot for BAF155 primary blot.

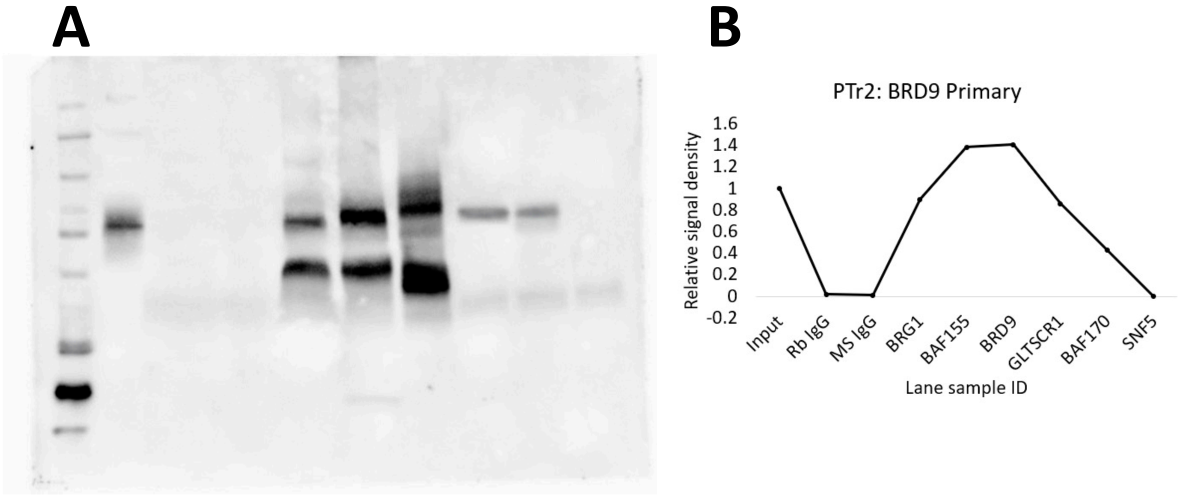

Figure S9. GBAF subunit BRD9 is detectable in PTr2 cells. (**A**) Co-IP of (starting in lane 5) BRG1, BAF155, BRD9, GLTSCR1, BAF170, and SNF5 with BRG1 primary antibody. (**B**) Accompanying relative signal density plot for BRD9 primary blot.

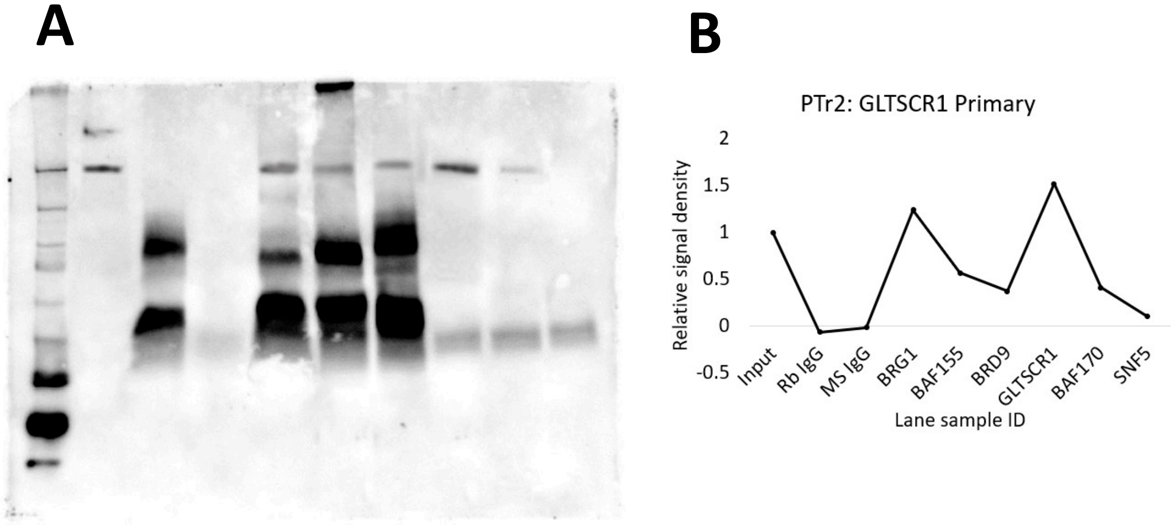

Figure S10. GBAF subunit GLTSCR1 is detectable in PTr2 cells. (**A**) Co-IP of (starting in lane 5) BRG1, BAF155, BRD9, GLTSCR1, BAF170, and SNF5 with BRG1 primary antibody. (**B**) Accompanying relative signal density plot for GLTSCR1 primary blot.

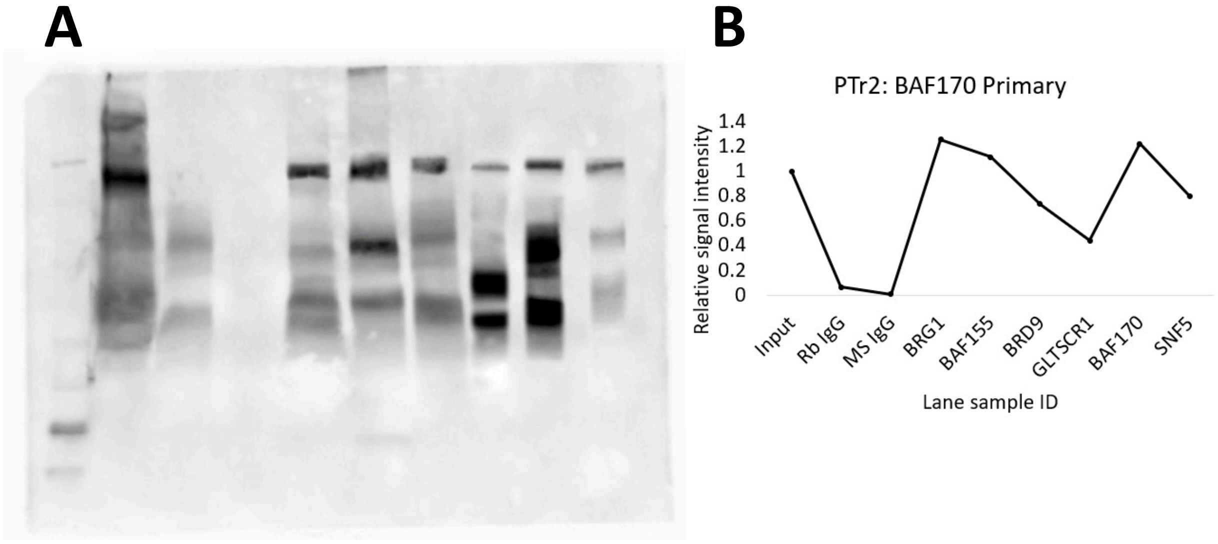

Figure S11. GBAF subunits BRD9 and GLTSCR1 are co-enriched with BAF170 in PTr2 cells. (**A**) Co-IP of (starting in lane 5) BRG1, BAF155, BRD9, GLTSCR1, BAF170, and SNF5 with BRG1 primary antibody. (**B**) Accompanying relative signal density plot for BAF170 primary blot.

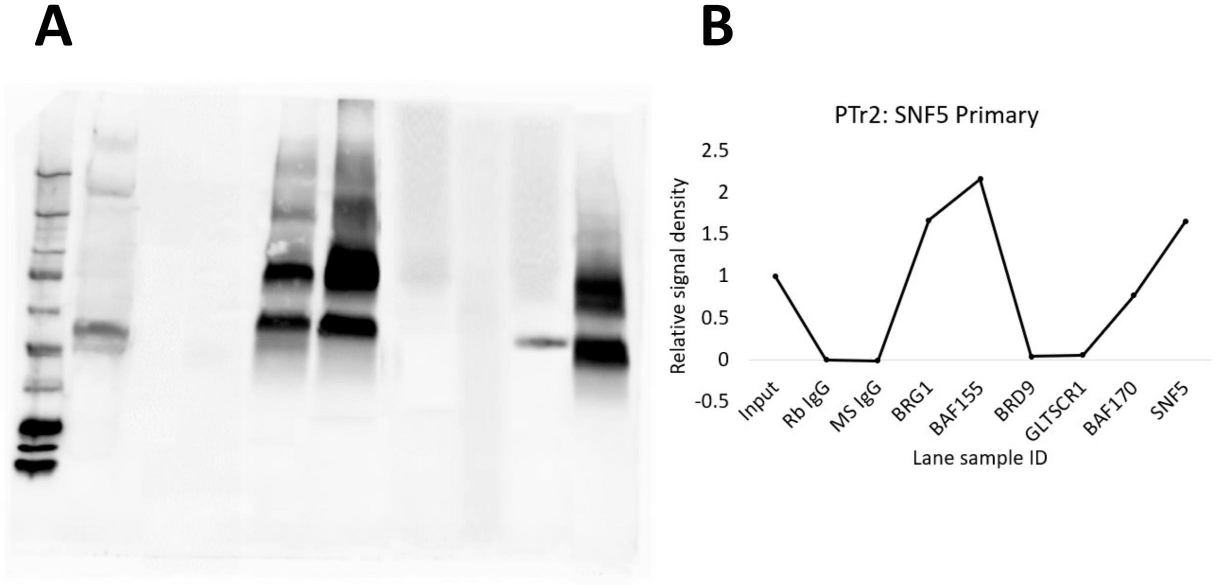

Figure S12. GBAF subunits BRD9 and GLTSCR1 are not co-enriched with SNF5 in PTr2 cells. (**A**) Co-IP of (starting in lane 5) BRG1, BAF155, BRD9, GLTSCR1, BAF170, and SNF5 with BRG1 primary antibody. (**B**) Accompanying relative signal density plot for SNF5 primary blot.
